# Emergency Admissions for Cardiovascular and Respiratory Diseases and the Chemical Composition of Fine Particle Air Pollution

**DOI:** 10.1289/ehp.0800185

**Published:** 2009-02-11

**Authors:** Roger D. Peng, Michelle L. Bell, Alison S. Geyh, Aidan McDermott, Scott L. Zeger, Jonathan M. Samet, Francesca Dominici

**Affiliations:** 1 Department of Biostatistics, Johns Hopkins Bloomberg School of Public Health, Baltimore, Maryland, USA; 2 School of Forestry and Environmental Studies, Yale University, New Haven, Connecticut, USA; 3 Department of Environmental Health Sciences, Johns Hopkins Bloomberg School of Public Health, Baltimore, Maryland, USA; 4 Department of Preventive Medicine, University of Southern California, Los Angeles, California, USA

**Keywords:** cardiovascular disease, chemical components, hospital admission, particulate matter, PM_2.5_, respiratory disease, Speciation Trends Network

## Abstract

**Background:**

Population-based studies have estimated health risks of short-term exposure to fine particles using mass of PM_2.5_ (particulate matter ≤ 2.5 μm in aerodynamic diameter) as the indicator. Evidence regarding the toxicity of the chemical components of the PM_2.5_ mixture is limited.

**Objective:**

In this study we investigated the association between hospital admission for cardiovascular disease (CVD) and respiratory disease and the chemical components of PM_2.5_ in the United States.

**Methods:**

We used a national database comprising daily data for 2000–2006 on emergency hospital admissions for cardiovascular and respiratory outcomes, ambient levels of major PM_2.5_ chemical components [sulfate, nitrate, silicon, elemental carbon (EC), organic carbon matter (OCM), and sodium and ammonium ions], and weather. Using Bayesian hierarchical statistical models, we estimated the associations between daily levels of PM_2.5_ components and risk of hospital admissions in 119 U.S. urban communities for 12 million Medicare enrollees (≥ 65 years of age).

**Results:**

In multiple-pollutant models that adjust for the levels of other pollutants, an interquartile range (IQR) increase in EC was associated with a 0.80% [95% posterior interval (PI), 0.34–1.27%] increase in risk of same-day cardiovascular admissions, and an IQR increase in OCM was associated with a 1.01% (95% PI, 0.04–1.98%) increase in risk of respiratory admissions on the same day. Other components were not associated with cardiovascular or respiratory hospital admissions in multiple-pollutant models.

**Conclusions:**

Ambient levels of EC and OCM, which are generated primarily from vehicle emissions, diesel, and wood burning, were associated with the largest risks of emergency hospitalization across the major chemical constituents of PM_2.5_.

Epidemiological studies have provided evidence of an association between short-term and long-term exposure to fine particulate matter [≤ 2.5 μm in aerodynamic diameter (PM_2.5_)] and risk for premature mortality and excess morbidity (Pope and Dockery 2006). These studies have focused on the risk associated with the total mass of particles, without considering the heterogeneity in their chemical composition. The U.S. National Research Council Committee on Research Priorities for Airborne Particulate Matter identified the assessment of PM characteristics associated with toxicity as a crucial research gap (National Research Council 2004), and many researchers have called for studies on possible differential toxicity of PM based on its components (Nel 2005). Toxicologic studies have linked different size ranges and constituents of PM with various health responses, but few of these results have been replicated by epidemiologic studies examining exposures at ambient concentrations (Schlesinger et al. 2006). Given the complex mixture of PM in ambient air, characterizing the biological mechanisms through which PM harms human health is challenging, especially because multiple mechanisms are likely to be operative across the range of health outcomes associated with exposure to PM. Currently, many hypotheses have been advanced (Brook et al. 2004; Pope and Dockery 2006; Schlesinger et al. 2006), but understanding of mechanisms is not yet sufficient to integrate what is known about the characteristics of PM in ambient air with the epidemiologic and toxicologic findings (Kaufman 2007).

Limited understanding of the differential toxicity of various components of the PM mixture also hinders control programs intended to reduce the public health burden of morbidity and mortality from airborne PM. Present measures to reduce the health effects of ambient PM depend largely on controlling the emission sources that contribute the most to PM mass. Promulgating more targeted air quality standards or guidelines incorporating PM chemical components, other characteristics, or specific sources of PM (e.g., traffic vs. electrical utilities) requires a more complete scientific foundation than is currently available.

The availability of PM_2.5_ component data from the U.S. Environmental Protection Agency (EPA) allows us to examine at a population level the heterogeneity in toxicity of PM_2.5_ components. In previous work we identified seven components of PM_2.5_ that contribute most of the PM_2.5_ total mass or that covary closely with PM_2.5_ total mass (Bell et al. 2007). We also reported evidence of an association between PM_2.5_ total mass and hospital admissions in the Medicare population for the period 1999–2005 for 204 U.S. counties (Dominici et al. 2006; Peng et al. 2008) and examined the associations of the coarse fraction of PM [> 2.5 and ≤ 10 μm in aerodynamic diameter (PM_10–2.5_)] with hospital admissions for 108 U.S. counties (Peng et al. 2008). For the present study, we used time-series approaches and estimated the association between the seven PM_2.5_ chemical components and risk of hospital admissions among Medicare enrollees for cardiovascular disease (CVD) and respiratory disease over the period 2000–2006.

## Methods

We obtained daily counts of hospital admissions for the period 2000–2006 from billing claims of enrollees in the U.S. Medicare system. Because the Medicare data analyzed for this study did not include individual identifiers, we did not obtain consent from individuals. This study was reviewed and exempted by the Institutional Review Board at the Johns Hopkins Bloomberg School of Public Health.

Each billing claim contains the date of service, disease classification using *International Classification of Diseases, 9th Revision* (ICD-9) codes (Centers for Disease Control and Prevention 2008), age, and county of residence. We considered two broad classes of outcomes based on ICD-9 codes: urgent or emergency cardiovascular admissions and urgent or emergency respiratory admissions. The classification of “urgent” and “emergency” is designated directly on each Medicare hospital admissions record. We excluded other classifications, such as “elective.” A recent study (Dominici et al. 2006) considered a number of different cardiovascular and respiratory outcomes. Because of the sparser sampling of the PM_2.5_ component data compared with the PM_2.5_ total mass data, to obtain sufficient statistical power we collapsed the data into two broad categories of hospital admissions: *a*) CVD, which includes heart failure (ICD-9 code 428), heart rhythm disturbances (426–427), cerebrovascular events (430–438), ischemic heart disease (410–414, 429), and peripheral vascular disease (440–448); and *b*) respiratory diseases, which include chronic obstructive pulmonary disease (490–492) and respiratory infection (464–466, 480–487). We excluded admissions for injuries and for external causes (800–849). By collapsing these health outcomes, we increased statistical power and obtained more stable estimates of risk at the cost of some specificity of the outcome.

We analyzed each outcome (respiratory or cardiovascular admissions) separately. We calculated the daily counts of hospitalizations by summing the hospital admissions for each disease of interest recorded as a primary diagnosis. To calculate daily hospitalization rates, we constructed a parallel time series of the numbers of individuals enrolled in Medicare that were at risk in each county on each day. We based the location of each hospital admission on the county of residence of the enrollee.

**Figure 1:**
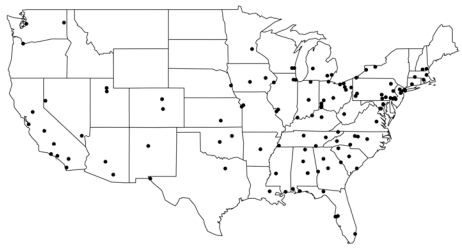
U.S. counties with populations larger than 150,000 for which sufficient hospital admissions and PM_2.5_ chemical component data were available, 2000–2006 (119 total).

The U.S. EPA established the PM Speciation Trends Network (STN) to measure more than 50 PM_2.5_ chemical components, in addition to total mass. The STN includes > 50 national air monitoring stations (NAMS) and > 200 state and local air monitoring stations (SLAMS) (U.S. EPA 1999). Air pollution concentrations were typically measured on a 1-in-3–day schedule in the NAMS and on a 1-in-6–day schedule in the SLAMS. We removed suspect data and extreme values from the original monitor records; monitors with very little data were omitted altogether. Full details of the construction of the database can be found else-where (Bell et al. 2007). We also used PM_2.5_ total mass measurements from the U.S. EPA’s Air Quality System as in our previous analyses (Dominici et al. 2006). Of the 187 counties described in the Bell et al. (2007) analysis, we restricted the present analysis to counties with general populations larger than 150,000 and with at least 100 observations on components of PM_2.5_. These requirements ensured that we would have enough data in a particular location to estimate an association between PM_2.5_ components and hospital admissions. The study population consisted of 12 million Medicare enrollees living in 119 urban counties in the United States (Figure 1).

We limited our analysis to the components making up a large fraction of the total PM_2.5_ mass or covarying with total mass (Bell et al. 2007): sulfate, nitrate, silicon, elemental carbon (EC), organic carbon matter (OCM), sodium ion, and ammonium ion. These seven components, in aggregate, constituted 83% of the total PM_2.5_ mass, whereas all other components individually contributed < 1%. We computed countywide averages for each of these components and for PM_2.5_ total mass by averaging the daily values from all monitors in a county. We adjusted organic carbon measurements for field blanks to estimate OCM. We used a standard approach such that OCM = *k*(OC_m_ − OC_b_), where OCM represents organic carbon matter, OC_m_ represents measured organic carbon, OC_b_ represents organic carbon for blank filters, and *k* is the adjustment factor to account for non-carbon organic matter. We applied a *k* value of 1.4, as in a previous analysis (Bell et al. 2007). We obtained temperature and dew-point temperature data from the National Climatic Data Center on the Earth-Info CD database (EarthInfo 2006).

As a check on the consistency of the chemical component data, we first assessed whether three different PM_2.5_ indicators (four scenarios total) provided comparable estimates of the short-term associations of PM_2.5_ with cardiovascular and respiratory admissions: PM_2.5_ (1), PM_2.5_ measured by the national PM_2.5_ monitoring network for the period 1999–2006; PM_2.5_ (1a), PM_2.5_ (1) for the period 2000–2006 and including only days with available measurements for all the seven PM_2.5_ components from the STN; PM_2.5_ (2), PM_2.5_ measured by the STN for the period 2000–2006 and including only days with available measurements for all the seven PM_2.5_ components from the STN; and PM_2.5_ (3), PM_2.5_ estimated as the sum of the seven largest components of PM_2.5_ mass for the period 2000–2006. Significant differences between these estimates would raise uncertainty as to the recorded values of PM_2.5_ total mass and its components. The estimates obtained under the scenarios 1a, 2, and 3 use data on the same subset of days. Each of these measures of PM_2.5_ was available in all 119 counties.

We estimated the within-county monitor-to-monitor correlation for each of the seven PM_2.5_ components to obtain a measure of the spatial homogeneity of each component. For this calculation we used a subset of 12 counties that had more than one monitor (27 monitors total): Jefferson, Alabama; Washington, DC; Cook, Illinois; Jefferson, Kentucky; Wayne, Michigan; Bronx, New York; Cuyahoga, Ohio; Allegheny, Pennsylvania; Philadelphia, Pennsylvania; Providence, Rhode Island; King, Washington; and Kanawha, West Virginia. We computed correlations only if at least 90 paired observations were available between two monitors. We also estimated the median within-county correlations between the seven PM_2.5_ components, and the three measures of PM_2.5_ total mass by *a*) estimating the correlations between time series data for each pair of air pollutants within each county and *b*) taking the median of the estimated correlations across the 119 counties. As a separate measure of spatial homogeneity, we calculated, for each of the seven components and using all monitors, the distance at which the correlation between pairs of monitors was 0.5 on average.

We applied Bayesian hierarchical statistical models to estimate county-specific and national average associations between daily variation in the seven PM_2.5_ chemical components and daily variation in hospital admissions rates. This approach was originally developed for the National Morbidity, Mortality, and Air Pollution Study (Bell et al. 2004; Samet et al. 2000) and subsequently extended (Dominici et al. 2006) to provide a consistent and unified methodology for analyzing data from multiple locations. We fit log-linear Poisson regression models with overdispersion to county-specific time-series data on hospital admissions and chemical components, accounting for potential confounders such as weather, day of the week, unobserved seasonal factors, and long-term trends. In each county-specific regression model, we included an indicator for the day of the week, a smooth function of time with 8 degrees of freedom (df) per calendar year to control for seasonality and long-term trends, a smooth function of current-day temperature (6 df), a smooth function of the 3-day running mean temperature (6 df), a smooth function of current-day dew-point temperature (3 df), and a smooth function of the 3-day running mean dew-point temperature (3 df). For all of the smooth functions we used a natural spline basis. We conducted a sensitivity analysis with respect to the smooth function of time to determine the degree to which risk estimates changed with varying levels of adjustment for smooth unmeasured confounders. Although other information about Medicare enrollees is available, such as sex and race, we excluded these factors from all models because they do not vary over time and should not play a role in our time series analysis.

For the exposure concentrations, we examined 0-, 1-, and 2-day lag concentrations because our previous work with PM_2.5_ total mass and hospital admissions showed little evidence of a strong association with admissions at a lag of ≥ 3 days (Dominici et al. 2006). We examined each lag separately because the 1-in-6–day sampling of the chemical component data from the STN prohibited the use of distributed lag models where all lags can be examined simultaneously.

For estimating the health effects of the PM_2.5_ components, we employed single-pollutant and multipollutant models. In single-pollutant models, we included each PM_2.5_ component in the regression model individually, without adjusting for any other chemical component (the model does adjust for other time-varying factors). In multipollutant models, we included PM_2.5_ components simultaneously to obtain estimates of the regression coefficients for each component adjusted for the other components. Ammonium was excluded from models that included sulfate and nitrate because of the high correlation among these three components. We included ammonium in a separate multipollutant model that did not include sulfate or nitrate but included the remaining four components.

We combined the county-specific risk estimates to form a national average using a Bayesian hierarchical model. In the single-pollutant models, we combined the log-relative risks separately for each pollutant using TLNise two-level normal independent sampling estimation software (Everson and Morris 2000). For the multiple-pollutant models, the risks were treated as a vector for each county and combined using a multivariate normal hierarchical model. We used Markov chain Monte Carlo methods to obtain the posterior distribution of the national average component effects. We assessed statistical significance by the posterior probability that the national average relative risk for a component was greater than zero. Values of the posterior probability > 0.95 were considered statistically significant (Dominici et al. 2006; Peng et al. 2008).

We evaluated whether the relative risks of each PM_2.5_ component in a multipollutant model were equal. In this analysis, the risks represent the percent increase in admissions associated with a 1-μg/m^3^ increase in each PM_2.5_ component in a multipollutant model. We assessed the evidence against equal component risks using a chi-square statistic applied to the national average estimates. We also estimated the posterior probability that the coefficient for a particular component was greater than the mean of the coefficients for the other components.

For statistical calculations we used R statistical software, version 2.7.0 (R Foundation for Statistical Computing, Vienna, Austria).

## Results

Table 1 summarizes median, interquartile range (IQR), within-county monitor-to-monitor correlations, and percent contribution for each of the seven components of the PM_2.5_ total mass. We also calculated these statistics for the three indicators of PM_2.5_ total mass. The IQRs calculated here are medians of the within-county IQRs for the 119 counties, which indicate typical daily variation for each chemical component. This study used a total of 134 monitors measuring PM_2.5_ chemical components in 119 counties. Counties included in the study ranged in size from 23 square miles (New York County, NY) to 9,203 square miles (Maricopa County, AZ), with a median of 581 square miles in area. The median daily rates of admission per 100,000 for CVD and respiratory disease were 19.1 and 6.9, respectively, and the corresponding IQRs were 10.4 and 6.4.

The PM_2.5_ components that constitute a large fraction of PM_2.5_ mass had high correlation within counties. The distance at which the correlation between pairs of monitors was 0.5 on average was 285 miles for sulfate, 302 miles for nitrate, 188 miles for silicon, 109 miles for EC, 209 miles for OCM, 9 miles for sodium ion, and 210 miles for ammonium.

Table 2 summarizes the estimated correlations between the daily concentrations of county-specific time series for pairs of components. Although in the atmosphere the particle composition includes compounds such as ammonium sulfate and ammonium nitrate, the measurement strategy of the STN measures ammonium, sulfate, and nitrate separately. Therefore, concentrations of sulfate and ammonium as well as of nitrate and ammonium are highly correlated, with mean correlation coefficients of 0.83 and 0.52, respectively. The correlation between concentrations of ammonium and nitrate is close to 0.95 in some counties on the West Coast.

**Figure 2:**
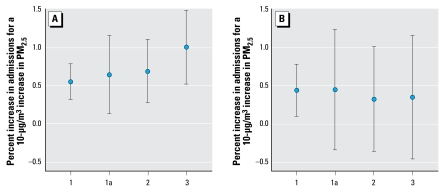
National average estimates and 95% PIs of the percent increases in emergency admissions for (*A*) CVD and (*B*) respiratory disease associated with PM_2.5_ at lag 0 under the four scenarios: PM_2.5_ (1), PM_2.5_ (1a), PM_2.5_ (2), and PM_2.5_ (3). The estimates obtained under scenarios 1a, 2, and 3 use data on the same exact subset of days.

We found that national average estimates obtained using the different measures of PM_2.5_ from the STN were generally consistent with previous findings, given the statistical uncertainties. Figure 2 shows the national average estimates and 95% posterior intervals (PIs) of the percent increases in emergency admissions for CVD (Figure 2A) and respiratory diseases (Figure 2B) associated with PM_2.5_ using three different indicators. Estimates obtained by restricting the data to days with available data from the STN (scenarios 1a, 2, and 3) have wider PIs because of the sparser sampling rate of the STN monitors. For scenario 1, on average, 1,896 days had observations for each county. For the remaining scenarios, on average, 410 days had observations for each county.

For the cardiovascular outcome, all four estimates show strong evidence of an association between PM_2.5_ and hospital admissions on the same day. Under scenario 1a, a 10-μg/m^3^ increase in PM_2.5_ is associated with a 0.64% (95% PI, 0.12–1.15%) increase, and under scenario 2, a 0.68% (95% PI, 0.26–1.10%) increase in hospital admissions for CVD. For respiratory outcomes, the evidence of an association between PM_2.5_ and hospital admissions was not as strong: under scenario 1a, a 0.44% (95% PI, –0.36 to 1.23%) increase in admissions, and under scenario 2, a 0.31% (95% PI, –0.38 to 1.01%) increase for a 10-μg/m^3^ increase in PM_2.5._ Although the point estimates are very similar, the uncertainty reflected in the PIs is much greater when restricted to days with measurements on the PM_2.5_ components from the STN (scenarios 1a, 2, 3). For both outcomes, lag 0 was specifically selected because previous work on PM_2.5_ total mass and hospital admissions showed that lag 0 exposure had the strongest association with cardiovascular and respiratory outcomes (Dominici et al. 2006).

**Figure 3:**
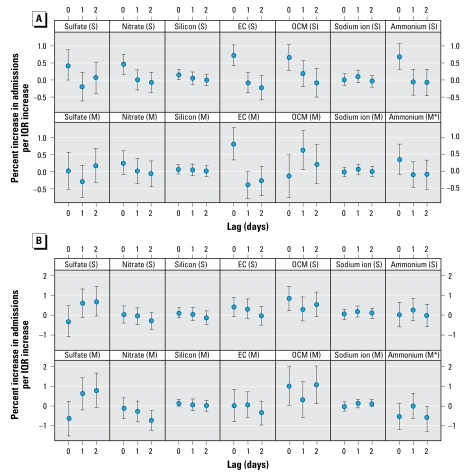
National average estimates and 95% PIs for the percent increase in hospital admissions for CVD (*A*) and respiratory disease (*B*) per IQR increase in each of the seven PM_2.5_ components in 119 U.S. counties during 2000–2006: single-pollutant model (S; top row) and multipollutant model (M; bottom row). The multipollutant model (M*) for ammonium excludes sulfate and nitrate.

Figure 3 shows national average estimates and 95% PIs for the percent increase in hospital admissions for CVD and respiratory diseases per IQR increase in each of the PM_2.5_ components, obtained from single-pollutant (top rows) and multipollutant models (bottom rows). Figure 3A indicates a positive and statistically significant association between CVD admissions and increases in nitrate, EC, OCM, and ammonium at lag 0 (same day) in single-pollutant models. We estimated that IQR increases in nitrate, EC, OCM, and ammonium were associated with 0.46% (95% PI, 0.17–0.75%), 0.72% (95% PI, 0.43–1.01%), 0.66 (95%% PI, 0.29–1.02%), and 0.68% (95% PI, 0.31–1.06%) increases in CVD admissions, respectively. In multipollutant models (Figure 3B), most risk estimates were reduced, unlike in the single-pollutant models. However, statistically significant associations remained, with an IQR increase in EC at lag 0 associated with a 0.80% (95% PI, 0.34–1.27%) increase in CVD admissions and an IQR increase in OCM at lag 1 associated with a 0.63% (95% PI, 0.06–1.19%) increase.

The association between respiratory admissions and OCM at lag 0 was statistically significant in single-pollutant models (Figure 3B). An IQR increase in OCM at lag 0 was associated with a 0.82% (95% PI, 0.22–1.44%) increase in respiratory admissions. In multipollutant models, we found statistically significant associations, with IQR increases in OCM at lag 0 and lag 2 associated with 1.01% (95% PI, 0.04–1.98%) and 1.07% (95% PI, 0.12–2.04%) increases in respiratory admissions, respectively.

We evaluated whether the health risks for each PM_2.5_ component in a multipollutant model were all equal. We found statistically significant heterogeneity in the toxicity of the components at lag 0 concentration for both the CVD and respiratory outcomes, indicating different degrees of toxicity. The Bayesian hierarchical model used here also provides a posterior probability distribution (given all of the observed data) for each of the risk parameters of interest. With this posterior distribution, we can calculate the posterior probability that a given estimate is larger than the average of the other five coefficients. For the CVD outcome, the posterior probability that the national average EC coefficient for lag 0 concentration was larger than the average of the other five coefficients in the multipollutant model was 0.99.

Sensitivity analysis indicated that results were not sensitive to changes in df for the smooth functions of time (ranging from 2 to 14 df per year), temperature, or dew-point temperature.

## Discussion

We conducted a multisite time series study to identify any major components of PM_2.5_ that were strongly associated with risk for hospitalization and found evidence of differing health risks across the components. We found that ambient levels of EC and OCM, which are generated primarily from vehicle emissions, diesel engines, and wood burning, were associated with the largest risk of emergency hospital admissions for CVD and respiratory disease in both single- and multiple-pollutant models. Furthermore, in multipollutant models we found evidence that the risk of cardiovascular admission associated with a same-day EC concentration was larger than the risks associated with any other component.

Our results add to those previously reported in several important ways. First, we used the largest database on chemical components of PM_2.5_ available on a national scale and found that ambient levels of EC and OCM were the most toxic components with respect to risk of emergency hospitalization for CVD and respiratory disease. These results, based on a large human population and using real-world concentrations of PM components, provide a much more complete picture of the health effects of PM chemical components than previously available. Second, the findings for EC and OCM, together with the weak evidence of harmful effects from the other components, substantially narrow the potential range of investigation and will aid the design of targeted mechanistic studies to test for parallel differential toxicity of these components in bioassays and to identify mechanisms of injury by these particular PM components. Such work may further lead to indications of appropriate biomarkers and the identification of sensitive subpopulations (Nel 2005). Third, this study indicates sources of PM air pollution that could be targeted as part of a comprehensive air quality control strategy. Because ambient particles are produced by numerous emission sources, any individual particle component identified as having an association with a health outcome may be acting as a marker for other components or a set of components with similar sources. For example, EC results from combustion of fossil fuels, including transportation sources such as diesel, but also from the combustion of biomass and coal and from industry (Health Effects Institute 1995). OCM also comes from vehicles, as well as from coal and oil combustion, industry, and vegetative burning. Finally, the statistical tools we have developed for this study represent a reproducible methodology that can be applied to other similar databases for studying the health effects of complex mixtures.

A few epidemiologic studies have investigated the potential toxicity of the PM_2.5_ chemical components on local or regional scales. In several of these studies, PM components have been examined as independent predictors, whereas one study used factor analysis of the Six Cities Study data to trace components back to their sources (Laden et al. 2000). Overall, the epidemiologic evidence linking particular PM components to health risks is mixed (Anderson et al. 2001; Burnett et al. 1997; Chuang et al. 2007; Dominici et al. 2007; Laden et al. 2000; Lippmann et al. 2006; Metzger et al. 2004; Ostro et al. 2007, 2008; Sarnat et al. 2006; Tolbert et al. 2000; Zanobetti and Schwartz 2006). Differences in findings may reflect the diversity of the study locations, health outcomes, or the analytic methods.

In our national study, cardiovascular admissions were most strongly associated with EC. Several other single-city or regional studies found associations between cardiovascular outcomes and EC. Ostro et al. (2007) found that risk of daily cardiovascular mortality increased by 2.1% (95% CI, 0.3–3.9) for a 0.8-μg/m^3^ increase in EC at a 3-day lag in a study of six California counties and, in a separate study, found that this association was stronger among people without a high school education (Ostro et al. 2008). Metzger et al. (2004) found statistically significant associations between EC and cardiovascular emergency department visits in Atlanta, Georgia, in single-pollutant models, as did Tolbert et al. (2000). These findings for EC point to a role for transportation sources in causing adverse health effects (Sarnat et al. 2008). Two source-directed analyses are similarly indicative: the Six Cities Study analysis by Laden et al. (2000) and a study of daily emergency room admissions in Boston (Zanobetti and Schwartz 2006). Lanki et al. (2006) examined five source indicators in data for Helsinki, Finland, and found that in multipollutant models, only EC was associated with ST segment depressions in elderly nonsmoking persons with stable coronary heart disease.

We also found evidence for association of OCM with respiratory admissions. In California, Ostro et al. (2007) found that the risk of cardiovascular mortality increased by 1.6% (95% CI, −0.1 to 3.2) per 4.6 μg/m^3^ increase in OCM with a 3-day lag but did not find strong associations with respiratory mortality. An association of OCM with cardiovascular emergency department visits was also found in Atlanta (Metzger et al. 2004; Tolbert et al. 2000).

EC and OCM were somewhat correlated on average (correlation = 0.64), and any reduction in effect size of either component when moving from the single-pollutant to the multipollutant model could be a result of this correlation. However, as with all results from multiple regression models, it is difficult to identify a single reason for changes in the risk estimates between the single-pollutant and multipollutant models. Correlation between pollutants in the model is one possibility, although not all of the pollutants examined here were highly correlated with each other. Another factor is the correlation of the pollutants with measured and unmeasured potential confounders, such as season and weather.

Ammonium ion was also strongly associated with cardiovascular admissions in single-pollutant models, although this association lost statistical significance when adjusted for other pollutants in the multipollutant model. The large effect observed in the single-pollutant model may result from another PM component that is correlated with ammonium ion. In addition, relatively little coherent evidence supports a role for ammonium ion and other secondary inorganic aerosols in causing adverse health effects (Schlesinger et al. 2006).

The evidence of increased risk of cardiovascular admissions and EC reported in this article is consistent with recent toxicologic findings. Biological responses have been shown to vary across characteristics and components of PM (Obot et al. 2004). Mechanisms by which inhaled particles may adversely affect cardiovascular and respiratory health have been proposed (Kendall et al. 2004; O’Neill et al. 2007), but specific mechanisms that might contribute to greater toxicity of particles with higher EC or OCM levels have not yet been identified (Pope and Dockery 2006). Toxicologic and epidemiologic studies have examined adverse health outcomes such as inflammation, oxidative stress, and alterations in cardiovascular function associated with particles (Schlesinger et al. 2006). In a study of young healthy adults, exposure to the OCM and EC components of concentrated ambient particles was linked with decreased brachial artery diameter (Urch et al. 2004). Associations have also been found between carbon black exposure and altered heart rate variability (Tankersley et al. 2004). The larger effect estimated for same-day EC is consistent with the literature on the biological mechanisms of CVD associated with short-term exposure to PM (Brook et al. 2004). Direct effects of PM can be associated with rapid cardiovascular responses such as increased myocardial infarctions and ischemia (e.g., Peters et al. 2001; Pope et al. 2006).

Measurement error due to a combination of instrument and laboratory error could affect our results because of biased measurements of the PM_2.5_ components. In particular, there is considerable uncertainty regarding the measurement of OCM because results depend on laboratory techniques and operational protocols (Kim et al. 2005). The U.S. EPA has conducted a quality assurance study of laboratories handling STN filter samples and the results indicate that, although there was some variability between laboratories (particularly for lower mass components, such as aluminum and sodium, and for specific subfractions of OCM), there was general consistency between the laboratories for most PM_2.5_ components (U.S. EPA 2005).

Exposure measurement error, which occurs when the monitor measurement of concentration is not representative of the exposure experienced by the study population, is also of concern. Our data and previous studies show that although PM_2.5_ total mass is relatively homogeneous within counties (Dominici et al. 2006), components with smaller contributions to total mass had lower within-county correlations. We would therefore expect that the countywide average for such components would be a poorer surrogate for personal exposure. To assess the potential effect of exposure measurement error for EC and OCM, we fit a separate measurement error model (Carroll et al. 2006) to adjust for spatial variability of each component [for details of the statistical models, see Supplemental Material (http://www.ehponline.org/members/2009/0800185/suppl.pdf)]. The national average associations of EC and OCM with cardiovascular and respiratory outcomes did not show qualitative differences. Another potential reason for exposure misclassification in this analysis is our restriction to more populous counties, which generally have substantial geographic areas. However, smaller cities (i.e., with < 150,000 residents) would likely produce risk estimates with greater uncertainties because of the smaller numbers of events. Hence, these estimates would be down-weighted when combined in our Bayesian hierarchical model and would likely contribute little to the overall results.

One limitation of this study is the 1-in-6–day sampling of PM_2.5_ chemical components dictated by the U.S. EPA’s monitoring schedule for the STN and other networks. Although we were able to estimate statistically significant short-term effects of single-lag concentrations, we were not able to fit distributed lag models that estimate cumulative short-term effects spread over multiple days. However, at this point the nature of this data set is a limitation to all researchers conducting national-level epidemiologic investigations of PM components. Although the national average estimates for a single lag might be an underestimate of the cumulative effect over a few days from the exposure, the single-lag effects estimated here nevertheless indicate a significant health risk from PM_2.5_ components.

Another limitation on the interpretation of our results is that our definitions of cardiovascular and respiratory admissions, which both include multiple ICD-9 diagnostic codes, reduce the specificity of the estimated associations compared with more narrowly defined disease categories. However, the benefit of our approach is the increased power to detect associations between chemical components and hospital admissions. Previous work (Dominici et al. 2006) examined the association between PM_2.5_ total mass and more specific CVD and respiratory disease categories and found that there was a strong association with all categories. Here, as in many studies, we had to strike a balance between power and specificity of the outcome.

Estimating effects of PM_2.5_ components is challenging because of their multiple and shared sources. We addressed this challenge by fitting multipollutant models to the largest national dataset available for the United States. EC and OCM, which we found to be the most injurious, are both found primarily in the fine fraction PM (PM_2.5_) as opposed to the coarse fraction (PM_10–2.5_). Our recent findings that PM_2.5_ is more injurious than coarse PM (Peng et al. 2008) is therefore consistent with the results reported here. The monitoring networks used in this study (the STN) are sited in or near urban communities, so the ambient exposures measured reflect the pollutant mix found in those communities. In addition, although we have made use of the U.S. EPA’s publically available national data on PM components, the geographic distribution of the monitors, determined by the U.S. EPA according to a variety of factors, is not uniform throughout the country. Hence, the sample of counties used in this analysis contains many counties from the industrial Midwest and Northeast regions of the country.

Although understanding the precise biological mechanism of injury from PM remains challenging, opportunities for developing targeted interventions for reducing ambient levels of PM air pollution are already apparent. Our results for EC and OCM suggest that the control strategies targeting their sources could be effective at reducing the public health burden attributable to PM. Given the U.S. EPA’s approach in the past to setting National Ambient Air Quality Standards, the evidence requirements will likely be high before any move toward standards or guidelines based on composition or sources can be made. This study, along with the related work of others, provides a substantial evidence base upon which further research into more refined air quality control strategies can be conducted.

## Figures and Tables

**Table 1:** Median and IQR values, within-county monitor-to-monitor correlations, and percent contribution for each of the seven components to the PM_2.5_ total mass, for 119 U.S. counties.

PM_2.5_ component	Median (μg/m^3^)	IQR (μg/m^3^)	Within-county correlation (min–max)	Percent PM_2.5_ mass
Sulfate	2.62	3.06	0.94 (0.77–0.99)	26
Nitrate	0.97	1.64	0.92 (0.78–0.99)	12
Silicon	0.07	0.07	0.63 (0.22–0.95)	1
EC	0.58	0.40	0.68 (0.42–0.85)	5
OCM	3.50	3.18	0.77 (0.53–0.93)	28
Sodium ion	0.09	0.11	0.43 (0.07–0.79)	1
Ammonium	1.18	1.35	0.91 (0.68–0.98)	11
Total mass				
PM_2.5_ (1)	11.79	9.38	0.85 (0.83–0.95)	
PM_2.5_ (2)	12.20	9.51	0.86 (0.73–0.99)	
PM_2.5_ (3)	10.40	8.09	0.86 (0.71–0.94)	

Abbreviations: max, maximum; min, minimum. Values are medians of the within-county values across all counties for the given statistic. Median (min–max) of the within-county monitor-to-monitor correlations of the seven components and of PM_2.5_ (2) and PM_2.5_ (3) were calculated using 27 monitors from 12 counties and only monitor pairs with more than 90 paired observations. Median and IQR (25th–75th percentiles) of the within-county monitor-to-monitor correlations of PM_2.5_ (1) using 401 monitors from 96 counties.

**Table 2:** Median (across counties) correlation between daily concentrations of time series for pairs of pollutants.

	Sulfate	Nitrate	OCM	EC	Silicon	Sodium ion	Ammonium	PM_2.5_ (1)	PM_2.5_ (2)	PM_2.5_ (3)
Sulfate										
Nitrate	0.09									
OCM	0.38	0.25								
EC	0.18	0.33	0.64							
Silicon	0.18	−0.04	0.22	0.14						
Sodium ion	0.10	0.12	0.10	0.03	0.10					
Ammonium	0.83	0.52	0.45	0.33	0.11	0.04				
PM_2.5_ (1)	0.76	0.39	0.68	0.46	0.22	0.07	0.85			
PM_2.5_ (2)	0.73	0.39	0.68	0.47	0.26	0.09	0.82	0.91		
PM_2.5_ (3)	0.75	0.48	0.78	0.55	0.20	0.13	0.88	0.92	0.91	

PM_2.5_ (1), PM_2.5_ (2), and PM_2.5_ (3) indicate different PM_2.5_ scenarios.
